# γ-Lindane Increases Microcystin Synthesis in *Microcystis aeruginosa* PCC7806

**DOI:** 10.3390/md13095666

**Published:** 2015-09-03

**Authors:** Laura Ceballos-Laita, Laura Calvo-Begueria, Jessica Lahoz, María-Teresa Bes, María F. Fillat, María-Luisa Peleato

**Affiliations:** 1 Department of Biochemistry and Molecular & Cellular Biology, and the Institute for Biocomputation and Physics of Complex Systems (BIFI), University of Zaragoza, Pedro Cerbuna 12, 50009 Zaragoza, Spain; E-Mails: laura_ceballos1@hotmail.com (L.C.-L.); laucalvobegueria@gmail.com (L.C.-B.); tbes@unizar.es (M.-T.B.); fillat@unizar.es (M.F.F.); 2 Laboratorio de Calidad de las Aguas, Confederación Hidrografica del Ebro, Plaza Canal Imperial, n° 9, 50004 Zaragoza, Spain; E-Mail: kekika1983@hotmail.com

**Keywords:** microcystin, lindane, *Microcystis*

## Abstract

HCH factories, and the waste dumpsites associated to its production, have become a global environmental concern, and their runoff could pollute ground and surface waters with high levels of the pollutant. In this study, the influence of lindane (γ-HCH) on microcystin production has been investigated in *Microcystis aeruginosa* PCC7806. This toxic cyanobacterium is highly tolerant to γ-lindane (20 mg/L), and produces more toxin (microcystin) in the presence of the pollutant. *Microcystis* degrades γ-lindane and presence of γ-lindane induces genes involved in its own degradation (*nirA*). RT-PCRsq has been used to monitor changes in levels of transcripts encoded by the *mcy* operon (*mcyD*, *mcyH* and *mcyJ*), responsible for the microcystin synthesis machinery, as well as other genes involved in its transcriptional regulation, such as *ntcA* and *fur* family members. The presence of lindane in the culture media induces *mcyD* expression, as well as *ntcA* gene transcription, while other genes, such as *mcyH*, (putative ABC transporter), are downregulated. The amount of microcystin found in the cells and the culture media is higher when *M. aeruginosa* is treated with γ-lindane than in control cells. The results suggest that in a lindane polluted environment, *Microcystis* toxic strains may enhance their microcystin synthesis.

## 1. Introduction

Cyanobacteria are organisms with an outstanding capacity to adapt to a wide range of environments and survive in extreme or highly degraded environments. Their metabolic plasticity includes the synthesis of a broad variety of secondary metabolites, some of them potentially toxic for eukaryotic organisms, the so-called cyanotoxins [1,2]. Moreover, changes in the environment, such as the eutrophication of aquifers, provoke blooms of cyanobacteria, in many cases producing high levels of toxins in the water and thus causing serious health and environmental problems [1]. *Microcystis aeruginosa* is a freshwater cyanobacterium responsible for many toxic blooms, producing microcystin. Sometimes it is naturally occurring in the tidal fresh estuaries and low salinity coastal areas, and there is an alarming increase of blooms in such mesohaline estuaries [3].

The cyclic heptapeptide microcystin is the most ubiquitous and abundant cyanotoxin on Earth [1]. Microcystins are synthesized by a mixed polyketide synthase/nonribosomal peptide synthetase system called microcystin synthetase. Tillett and co-workers [4] identified and sequenced the gene cluster named *mcy* operon, which encodes the microcystin synthetase machinery in *Microcystis aeruginosa* PCC7806. This 55-kb sequence consists of ten open reading frames transcribed bidirectionally from a central 732-bp intergenic region between *mcyA* and *mcyD*. Microcystin synthesis is an inducible event, which is probably regulated by many environmental and nutritional factors [5].

The extreme adaptability of some cyanobacteria makes them able to tolerate and even metabolize moderate doses of pesticides [6,7]. Organochlorines, such as lindane (γ-hexachlorocychlohexane), are one of the most recalcitrant and persistent pesticides (Persistent Organic Pollutants, POP. Stockholm Convention 2009), having been used intensively in recent decades. Lindane was severely restricted in the 1970s and 1980s in the majority of developed countries [8], but is still used in some developing countries in Asia, Africa and Latin America [8]. Due to its long persistence in waters, lindane is now a widespread contaminant in aquatic ecosystems [9]. Several microorganisms have metabolic mechanisms to degrade lindane. Kuritz *et al*. [6] highlight the ability of fifteen strains of cyanobacteria to degrade lindane. This process seems to be dependent on a functional *nir* operon, involved in the nitrite reduction system, implicated in a dechlorination process [10]. Cyanobacteria show a remarkable potential in the bioremediation of aquatic and terrestrial habitats [11], but the risks of proliferation of potentially toxic strains or the increasing toxin production in the presence of contaminants require rigorous evaluation. It has been shown that ampicillin, atrazine and cadmium downregulate the transcription of microcystin synthesis gene clusters [12], demonstrating that the aforementioned antibiotics, herbicides and heavy metals affect microcystin levels. A review of the available literature shows ample data variations both in lindane tolerance and lindane degradation depending on the cyanobacterial strain used [6,11,13,14]. Lindane physiological effects on growth (decreased), photosynthesis (decreased) and oxidative metabolism (antioxidant defenses increased) have been described in non-toxic strains of *Anabaena* (PCC7120 [14] and in a strain isolated from paddy fields [13].

In Spain, as well as around the world, there are several lindane residues dumpsites, with high levels of the pollutant, a consequence of former lindane factories activity. Risk management of eventual leachates of dumpsites requires knowledge of secondary environmental problems derived from the presence of lindane. In this study, the influence of lindane (γ-HCH) on microcystin production has been investigated in *M. aeruginosa* PCC7806 exposed to the pesticide. The changes in levels of transcripts encoding *mcyD*, *mcyH*, and *mcyJ* in treated cells have been determined using semi-quantitative RT-PCR. The expression of the transcriptional regulators involved in *mcy* operon regulation has also been examined together with *nirA*, since lindane degradation depends on an active *nir* operon [10].

## 2. Results and Discussion

### 2.1. Effects of Lindane on M. Aeruginosa PCC7806 Growth

**Figure 1:**
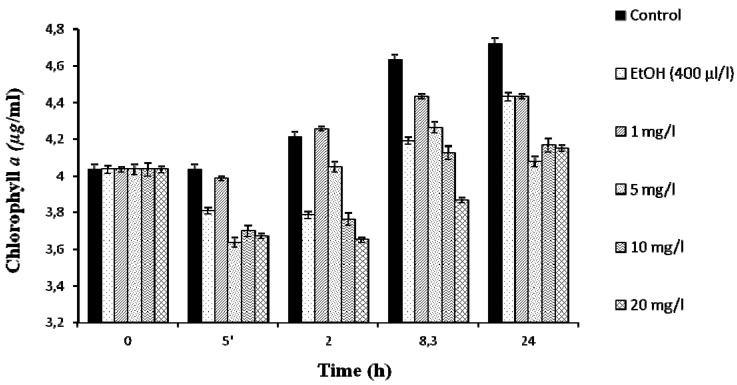
Survival of *M. aeruginosa* PCC7806 cells grown in the presence of different amounts of γ-lindane, dissolved in 400 µL/L of ethanol.

**Figure 2:**
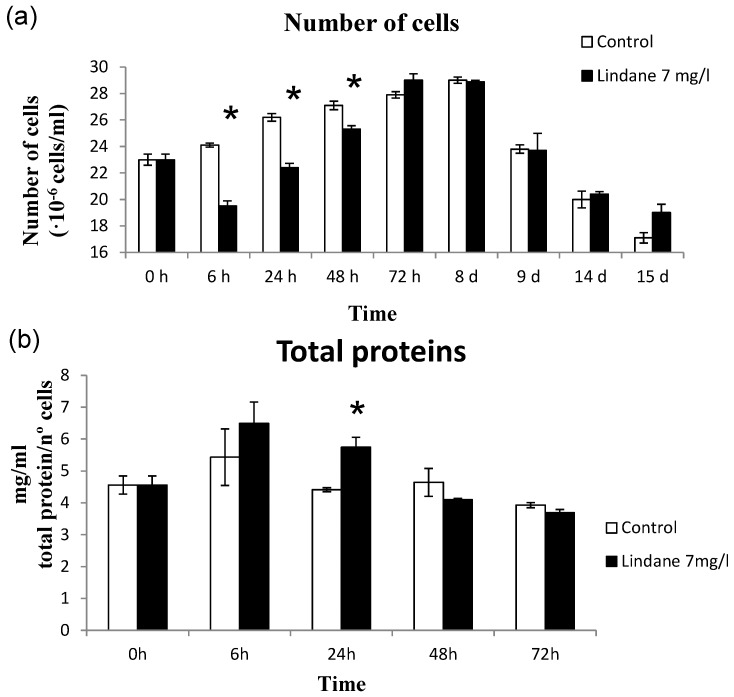
Effect of lindane. (**a**) Growth of *M. aeruginosa* PCC7806 in the presence of lindane at its limit of solubility in water, 7 mg/L; (**b**) Total protein determined in cells growing in 7 mg/L lindane. * significant *p* < 0.05.

The toxic *Microcystis aeruginosa* PCC7806 exhibits a considerable tolerance to lindane, surviving and growing up to 20 mg/L (Figure 1), whereas the growth of a field isolated *Microcystis aeruginosa* strain (Kützing) Lemmermann (MaD7) was completely inhibited at 5 mg/L [11]. Initially, lindane was added from a stock solution in ethanol. The presence of 0.04% ethanol, as used for lindane solubilization in most published papers (5% ethanol in [11], 0.1% in [14]), affects the growth of the cyanobacteria (Figure 1). For this reason, in subsequent experiments, lindane was used at its limit of solubility in water, 7 mg/L. Growth curves were determined and the results are shown in Figure 2. The data indicates that lindane affects cell survival and growth, but after a few hours a certain recovery could be observed. The recovery could be due either to the adaptation of the *Microcystis* cells to the pesticide or/and the induction of the metabolic mechanism to metabolize moderate amounts of lindane. Initially, cells increased their protein content as a response to lindane, but after 48 h, control and lindane-treated cells showed similar levels.

**Figure 3:**
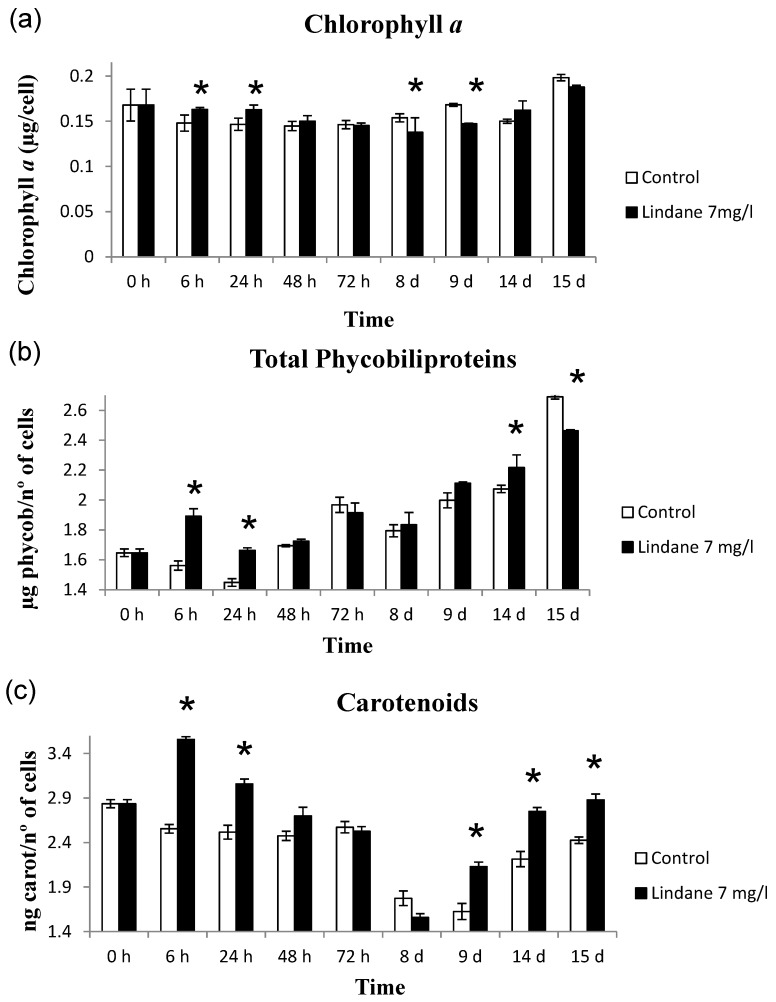
Effect of lindane on the pigment content in *M. aeruginosa* PCC7806. (**a**) chlorophyll *a*; (**b**) phycobiliproteins; (**c**) carotenoids. * Significant *p* < 0.05.

**Figure 4:**
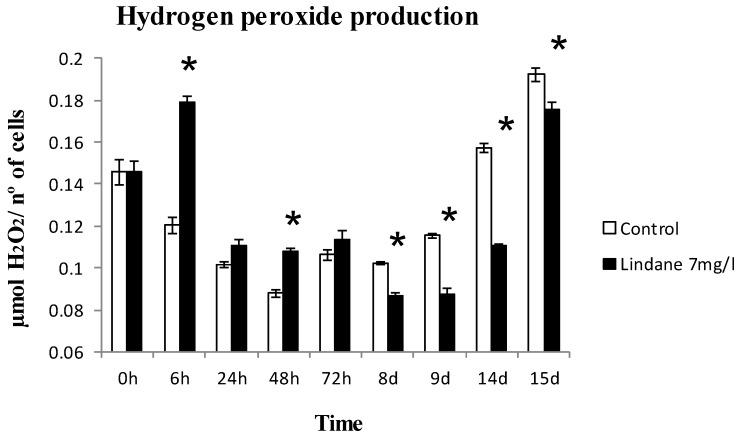
Generation of H_2_O_2_ in *M. aeruginosa* PCC7806 cells under lindane treatment. * significant *p* < 0.05.

Chlorophyll *a*, phycobiliproteins and carotenoids were determined and the results are shown in Figure 3. When exposed to lindane, the amount of cell pigments is not altered in the case of chlorophyll *a*, whereas synthesis of carotenoids and phycobiliproteins increased (Figure 3). H_2_O_2_ production increase was found in early stages of the lindane treatment (Figure 4).

### 2.2. Effects of Lindane on Gene Expression Levels

The *mcyD* gene, transcribed from the bidirectional promoter present in the *mcy* cluster encodes a modular polyketide synthase involved in the synthesis of Adda, the β-amino acid responsible for the toxicity of all variants of microcystins. Moreover, *mcyD* expression is essential in microcystin synthesis and the lack of the protein results in the absence of microcystin synthesis [5]. For these reasons we considered it a suitable gene candidate to be studied as *mcy* expression marker.

**Figure 5:**
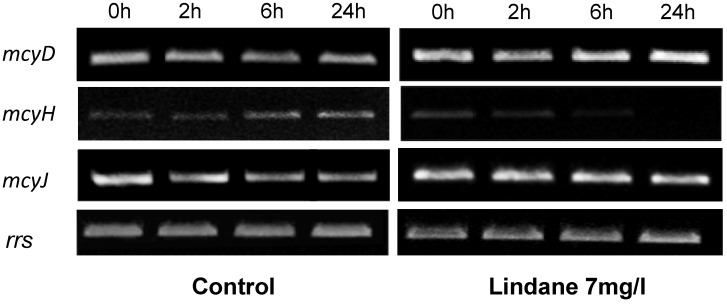
Changes in the *mcyD*, *mcyH* and *mcyJ* expression in *M. aeruginosa* PCC7806 cells treated with lindane. The expression was studied with semi-quantitative RT-PCR. The housekeeping gene *rrs* was used as control. Determinations for each gene were performed in the exponential phase of PCR. Experiments were repeated at least 3 times with independent RNA extractions and the relevant portions of a representative gel are shown.

Aliquots of those cells growing in 7 mg/L lindane were studied *versus* non-stressed cells. *M. aeruginosa* PCC7806 cells were grown at 40 μmol photons m^−2^·s^−1^ until they reached an early exponential phase of growth (OD_700 nm_ = 0.6). Lindane was then added to the culture. Aliquots were collected at different times and the total RNA was extracted and reverse-transcribed. RT-PCR was performed with cDNAs using specific primers for each gene, and a 16S rRNA gene was used as a housekeeping gene. The change in mRNAs levels in stressed cells was measured at different times and the data obtained are shown in Figure 5. *mcyD* is induced in lindane-treated cells. On the other hand, the data shown in Figure 5 indicate that *mcyH*, encoded for a putative ABC transporter protein, is downregulated in lindane-treated cells, with an apparent disappearance of the transcript. *mcyJ*, a putative methytransferase, increased very slightly its expression (Figure 5).

**Figure 6:**
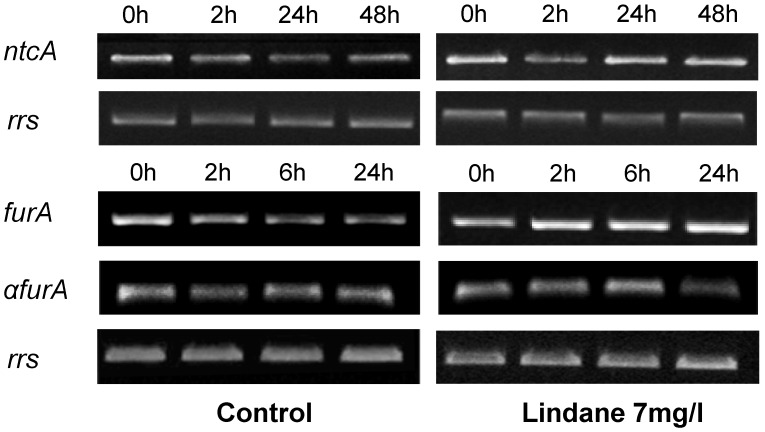
Semi-quantitative RT-PCR analysis of the expression of several genes involved in the regulation of the *mcy* operon. The housekeeping gene *rrs* was used as control. Determinations for each gene were performed in the exponential phase of PCR. Experiments were repeated at least twice with independent RNA extractions, and the relevant portions of a representative gel are shown.

*ntcA* and *fur*, genes corresponding with the two transcriptional regulators involved in *mcy* regulation [15,16], were also analyzed transcriptionally and the results obtained are indicated in Figure 6. *ntcA*, encoding the master regulator of the nitrogen metabolism, is clearly induced in the presence of lindane in comparison with control cells. The *furA* transcript level rises whereas its antisense, the *a-furA* [17], decreases, with a presumably final effect of more FurA protein synthesis. Other members of the Fur family, such as *furB* or *furC*, do not alter their expression upon treatment.

### 2.3. M. aeruginosa Degrades Lindane

Seven milligrams per liter of lindane was added to a culture of *M. aeruginosa* and to an identical volume of culture media without cells. After 1 and 15 days, aliquots were obtained and lindane concentration determined. Table 1 shows the observed changes. Cells cultured without lindane, as well as culture medium with the pollutant, were used as negative control. When *M. aeruginosa* is present, lindane concentration dropped after 15 days 6 times more than lindane in culture medium without cells (0.36 mg/L).

**Table 1:** Lindane presence in *M. aeruginosa* cultures as well as in control samples, 7 mg/L of lindane was added at time zero.

Sample	Time 24 h mg/L lindane	Time 15 days mg/L lindane
*M. aeruginosa* culture without lindane	<0.005	<0.005
Culture media 7 mg/L lindane	6.27 ± 0.34	2.08 ± 0.26
*M. aeruginosa* culture 7 mg/L lindane	6.19 ± 0.29	0.36 ± 0.05

**Figure 7:**
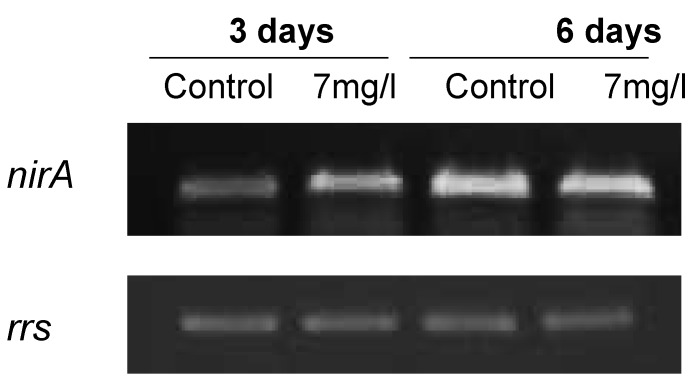
Transcriptional analysis of the *nirA* gene expression in lindane treated cells, using *rrs* as housekeeping reference.

Since an active *nir* operon is necessary for lindane dechlorination [10], the expression of *nirA* was also studied. The cells exhibit a considerable increase at 3 days of the *nirA* transcript in lindane-treated samples (Figure 7).

### 2.4. Microcystin-LR Content Correlates with mcyD Transcriptional Changes Caused by Lindane

**Figure 8:**
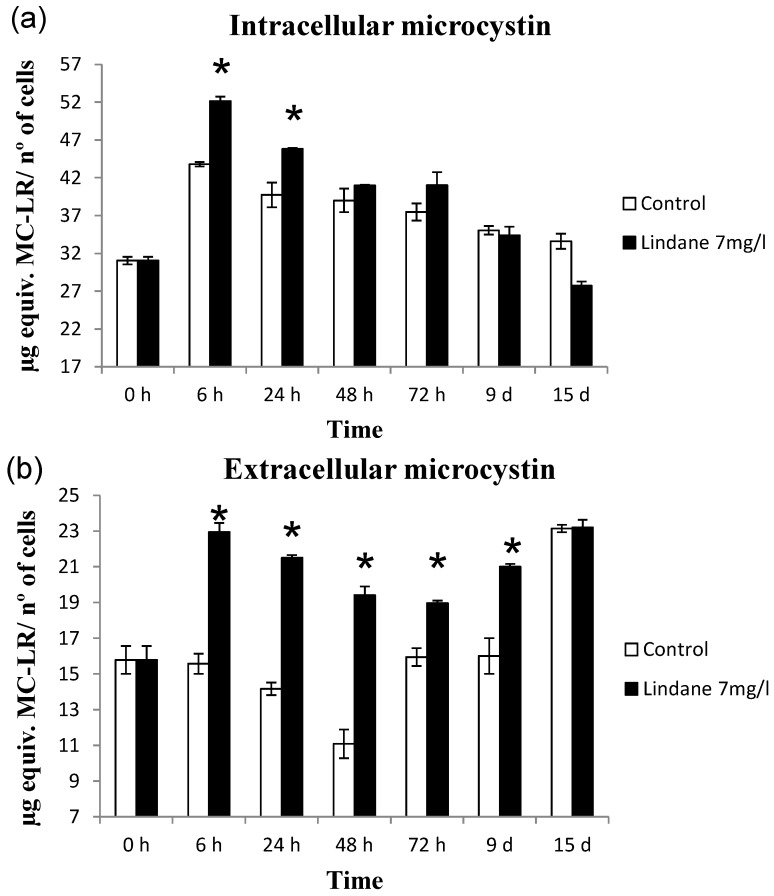
Microcystin-LR equivalents determined using MicroCystest^®^. (**a**) Intracellular microcystin content determined in lindane-treated cells and control cells; (**b**) extracellular microcystin content in lindane-treated cells and control cells. * Significant *p* < 0.05.

The increase of microcystin-LR content in the cells exposed to lindane was consistent with the changes found during the transcriptional analysis of the *mcyD* gene. Microcystin-LR (and the very low amount of D-Aps3 MC-LR, the only two variants found in this strain [18]) were determined using the MicroCystest^®^. The results expressed as microcystin-LR equivalents are represented in Figure 8. Figure 8A shows the microcystin present inside the cells while Figure 8B represents the microcystin extruded to the media. Lindane induces the synthesis of microcystin in *M. aeruginosa* and the increase coincided in time with the early gene induction responses to the pesticide. There was also an increment in the extracellular microcystin, probably due to the cell death observed as a consequence of lindane addition. In this case, the extracellular microcystin increase cannot be related to mcyH expression, which decreased in the presence of lindane.

### 2.5. Discussion

Cyanobacteria are considered as key primary producers in aquatic ecosystems, and there is a growing interest in how pollutants affect the physiology of phytoplankton. Moreover, the ability of several strains to produce cyanotoxins leads to the need to investigate if changes in the environment could affect this type of secondary metabolism. Lindane is one of the most widespread contaminants in aquatic environments and its impact on phytoplankton has been widely described (growth rate affectation, photosynthesis decrease and oxidative stress increase [11,13], though there are no references concerning its effect on cyanotoxin production. The amount of γ-lindane used in this work exceeds the usual levels found in rivers as a consequence of its pesticide use, but leachates from dumpsites can reach such concentrations [19].

After the addition of lindane, part of the population dies, but after a few hours and depending on the dose, growth is observed. As previously mentioned, it seems that *M. aeruginosa* PCC7806 tolerates higher lindane concentrations than other cyanobacteria reported in the literature, such as *Anabaena* PCC7120 [14] or other microcystin-producing strains such as *M. aeruginosa* (MaD7) or *Pseudoanabena limnetica* [11]. Additionally, *M. aeruginosa* PCC7806 seems to have the ability to degrade lindane, with the *nir* gene induced. Thus, this cyanobacterium can survive in a lindane-contaminated environment, and its tolerance may cause imbalances in the population of aquatic ecosystems, favoring the growth of this toxic strain. Lindane causes oxidative stress, but microcystin synthesis is not induced under such conditions [20]. Even though other microcystin-producing strains [11] do not tolerate more than 5 mg/L of lindane, according to Zilliges *et al.* [21], the presence of microcystin may increase the fitness of the cells under oxidative stress conditions.

Lindane increases slightly the *mcyD* transcript level (Figure 5), and the microcystin content per cell (Figure 8) correlates with the transcription of *mcyD* in all the experiments performed. Other pollutants such as ampicillin, atrazine and cadmium have been shown to decrease the *mcyD* transcript level in *M. aeruginosa* (strain: code 905 from the Institute of Hydrobiology, Chinese Academy of Sciences) [12], as well as the microcystin cell content. These authors also found *mcyH* downregulation, congruent with our experimental data where *mcyH* decreases slightly. McyH is a putative ABC transporter, and its inactivation results in loss of microcystin production [22]. Intracellular microcystin arises at the same time that *mcyH* is downregulated, but the more interesting point is in the change in the extracellular amount of toxin (Figure 8B). This microcystin can be released either from the initial cell death observed in Figure 1 and Figure 2, or because lindane could alter the cell wall and the membrane permeability. Observation of the cells by optical microscopy and scanning electron microscopy did not enable any apparent difference in size, surface or shape to be observed (data not shown).

*ntcA* increased its expression as a consequence of the presence of lindane. This key nitrogen transcriptional regulator exhibits affinity for two fragments of the bidirectional *mcyDA* promoter and 2-oxoglutarate enhanced its affinity, suggesting that the C to N metabolism balance regulates the microcystin gene cluster [15]. The increase in *ntcA* transcript can be explained from different points of view. Microcystin synthesis diverts a considerable amount of nitrogen, and the expression of NtcA can be a response to the stress of a lack of nitrogen available for the GS-GOGAT cycle. An increase in NtcA will induce the machinery for the uptake of nitrogen from the media. Other studies have shown that the degradation of lindane in other cyanobacteria was faster and more efficient in the presence of nitrogen [6] and the cell induces the machinery for its intake. On the other hand, *nirA* expression is enhanced in lindane treated *M. aeruginosa*. Since the *nir* operon is supposed to be involved in lindane dechlorination [10], the increase may be indicating that lindane is being degraded. Other transcriptional regulators from the Fur family involved in *mcy* operon regulation gave different results: *furB* and *furC* are not affected (data not shown). The increased amount of *furA* transcript and the decreased *a-furA* may again suggest that the cells are under oxidative stress from lindane exposure [17], congruent with the hydrogen peroxide increase (Figure 4), since there is no reason to think that the cells are iron-deficient.

## 3. Experimental Section

### 3.1. Growth Conditions

The axenic strain *Microcystis aeruginosa* PCC7806 was provided by the Pasteur Culture Collection (Paris, France) and grown in BG11 media [23] with 2 mM of NaNO_3_ as indicated by the Pasteur Institute. Cells were grown in batch conditions with continuous agitation at 25 °C. The cyanobacteria were grown using a light intensity of 40 µmol of photons m^−2^·s^−1^, unless indicated. Light was measured using a Quantum Sensor photometer (Skye Instruments, SKP 200). Every culture, control and stressed cell was started with equal aliquots of 0.3 OD (700 nm). When they reached the exponential growth phase (OD 700 nm, 0.6–0.7), they were treated as indicated in each experiment. Experiments were performed in duplicated cell culture of 250 mL in 500 mL Erlenmeyer flasks to avoid substantial volume changes during the sampling. Three different experiments were performed.

### 3.2. Analytical Methods and Microcystin Quantification

Samples of 1 mL and 5 mL were collected for chlorophyll *a* and protein determination. Chlorophyll *a* was extracted with 80% methanol and determined spectrophotometrically, quantified in accordance with the procedure described by Mackiney [24]. Total proteins were quantified according to the bicinchoninic acid (BCA) method (BCA^™^ Protein Assay Reagent Kit from Pierce) as previously described [25]. For the microcystin analysis, aliquots of cells (5 mL) were collected and frozen at −20 °C. Samples were taken out with 4 extractions after stirring for 10 min with 80% methanol, 0.1% trifluoroacetic acid (TFA), 0.1% tween and centrifugate at 4000 *g* for 10 min. The supernatant were pooled, and microcystin quantified using the MicroCystest^®^, as microcystin-LR equivalents (manufacturer’s information, based on protein-phosphatase inhibition). Carotenoids, phycobiliproteins, and H_2_O_2_ production were determined using 5 ml according to previously described methods [26,27,28].

Lindane was removed from the sample by extraction from water by adsorption on a magnetic bar stirrer of polydimethylpolysiloxane (SBSE) and analyzed by gas chromatography and mass spectrometry (GC/MS).

### 3.3. Cell Counting

The cell density was determined daily both spectrophotometrically and by counting cells with a Neubauer cell. To avoid cell aggregation, the samples were treated with KOH following the Azevedo methodology [2].

### 3.4. Sampling and RNA Isolation

Sampling was performed very carefully to avoid RNA degradation during manipulation. Aliquots of the cultures (25 mL) were harvested by centrifugation at 4000 *g* for 4 min at 4 °C. After removing the supernatant, each cell pellet was resuspended in 600 µL of 50 mM Tris-HCl (pH 8), 100 mM EDTA and 130 µL of chloroform and incubated in ice for 3 min to eliminate external RNases. The buffer was removed by centrifugation at 13,000 *g* for 5 min at 4 °C. Finally, the cell pellets were frozen in liquid nitrogen and kept at −80 °C until RNA isolation was achieved. Cells were lysed using TRIZOL (Invitrogen) according to the manufacturer’s instructions. After the chloroform extraction, RNA was collected from the aqueous layer and precipitated in isopropanol and liquid nitrogen. The RNA pellet was washed twice with 75% ethanol.

### 3.5. Reverse Transcription *(*cDNA Synthesis*)*

Prior to RT-PCR, the total RNA was treated with 40 units of DNase I (Pharmacia) in a volume of 100 µL using a buffer containing 4 mL of 1 M Tris-HCl (pH 7.5) and 0.6 mL 1 M of MgCl_2_ in DEPC-H_2_O. The sample was incubated at 37 °C for 1 h. After digestion, the enzyme was inactivated with one extraction of phenol:chloroform and RNA was precipitated with absolute ethanol. The successful digestion of DNA was assessed by PCR with primers targeting the16S rRNA gene (Table 2). RNA integrity was checked using a 1% agarose gel and the concentration was determined by measuring the absorbance at 260 nm. Its purity was assessed by the ratio A260 nm/A280 nm. For reverse transcription, 1 µg of total RNA was mixed with 150 ng of random hexamer primers (Invitrogen Corp.) and diluted with the annealing buffer (10 mM Tris-HCl (pH 8), 1 mM EDTA, 150 mM KCl) to a final volume of 10 µL. The mixture was heated at 85 °C for 10 min and then incubated at 50 °C for 1 h. After that, the RNA was reverse transcribed with 200 U of SuperScriptTM (GibcoBRL) in the presence of 2 µL of deoxyribonucleoside triphosphate mixture (2.5 mM each), 2 µL of dithiothreitol (100 mM) and 4 µL of the 5× buffer provided by the manufacturer with the Reverse Transcriptase enzyme kit. The volume was adjusted to 20 µL in DEPC-H_2_O. The mixture was incubated at 47 °C for 1 h and finally heated at 75 °C for 15 min.

**Table 2:** Oligonucleotides used as primers (RT-PCR).

Primers	Sequence 5′ → 3′	Length	Tm (°C)
*R16S* dir	CAAGTCGAACGGGAATCTTC	20	47
*R16S* rev	CTCAAGTACCGTCAGAACTTC	21	
*mcy D* dir	GAGCATTAAGGGCTAAATCG	20	45
*mcy D* rev	CTTGGTTGCTTCATCAACTC	20	
*mcy J* dir	GCCGAAGAAACAACTTATAACG	22	48
*mcy J* rev	CTATAGCCAAGCTTCCACCGGG	22	
RT-*mcy H* up	GGTATGAATGCAGCAG	16	45
RT-*mcy H* dw	CGCCTGGTTCGATAGG	16	
*ntcA* up	GGAATTCCATATGGACTTATCATTAATACAAGATAAAC	38	54
*ntcA* dw	CCCAAGCTTTTAAGTAAATTGTTGACTGAGAG	32	
*furA* dir	GTCGATCGCCCATGGCTGCCTAC	23	65
*furA* rev	CAGTTGGGAATTCCCGCTAGATG	24	
*αfurA* dir	CGACGATTTACCGCAGTG	18	53
*αfurA* rev	CACACTGTTTGAGACTGTG	19	
*nirA* dir	TGCCCATTCTACTCAACCCTA	21	58
*nirA* rev	GTGTCGCTAATCCCCATAGTTG	22	
*fur B* myc1 dir	CAATCTATGGGWYTAGCTACYGT	23	60
*fur B* myc1 rev1	CCGCAIARWCCAAAAAATTCIARIGTATG	29	
*fur C* myc dir	CATYTITCTGCTMGIGAAATTTATGATCC	29	60
*fur C* myc rev	CATGIGAATGIGAATCIGAAATAKTWCC	28	

### 3.6. RT-PCR Analysis of Gene Expression

Semi-quantitative RT-PCR assays were performed with the cDNA samples. Specific primer sets were designed to amplify both the studied genes and *16S rRNA* (*rrs*) housekeeping gene (Table 2). The exponential phase of each gene amplification reaction was estimated by measuring the amount of PCR products after different numbers of cycles, and the endogenous reference gene *rrs* was used to normalize the possible variation in cDNA concentration, as previously described [29]. PCR-amplified DNA fragments were observed by agarose gel electrophoresis 1%, stained with ethidium bromide and analyzed using a Gel Doc 2000 Image Analyzer (Bio-Rad). Samples for at least 3 different *Microcystis* growth curves were analyzed, with congruent results.

## 4. Conclusions

The results in this paper suggest that in a lindane polluted environment, *Microcystis aeruginosa* may enhance its toxicity, producing more microcystin. On the other hand, *Microcystis* shows capability to degrade γ-lindane and tolerate high concentration of the pollutant. Those facts are of interest first for environmental risk prevention and secondly, they open an interesting research area in bioremediation, with potential use of non-toxic *Microcystis* strains in lindane-polluted waters. However, the work presented here corresponds to laboratory data, and cannot be simplistically extrapolated to the natural environment.
